# Single Surgeon Experience With Minimally Invasive AVR: Expectations of a Change in Practice

**DOI:** 10.1186/1749-8090-10-S1-A120

**Published:** 2015-12-16

**Authors:** Larson Erb, Hany Abdallah, Turner Osler, Nkem Azikem, Annie Penfield-Cyr, Joseph Schmoker

**Affiliations:** 1 The University of Vermont Medical Center, Burlington, VT, USA

## Background/Introduction

Reports of minimally invasive aortic valve replacement (miniAVR) are hampered by non-standardized techniques and multi-surgeon/institution practice.

## Aims/Objectives

This study reports single surgeon/institution experience with standardized technique for AVR/miniAVR in all patients.

## Method

Retrospective chart review of entire experience of a single surgeon with isolated AVR or AVR/ascending aortic replacement. Conventional median sternotomy (cAVR, n = 144) was performed from 10/1998-10/2010 and compared after a change in practice to miniAVR (6 cm incision, ministernotomy J'ed into 3rd right intercostal space, n = 147) from 11/2010-12/2013. Four patients had planned cAVR after 11/2010 because of concerns on preoperative imaging and are included in the cAVR group. A propensity score matching model was used to reduce the impact of confounding bias in this retrospective observational dataset.

## Results

MiniAVR patients were older (mean age 70 vs. 66, p = 0.02) and had lower preoperative Hct (34% vs 38%, p < 0.001). There were more redo operations in the cAVR group (15% vs 8%, p = 0.03). There were no differences in other preoperative variables, including calculated STS mortality risk (3% miniAVR vs 4% cAVR). Operative/postoperative results are reported below (Table 1). There was no difference between groups in rate of stroke, MI, pneumonia, transfusion, inhospital mortality, 30 day mortality, and length of stay. Operative conversion rate to cAVR was 1%. There was a 26% absolute reduction in the rate of post-operative atrial fibrillation in the miniAVR group corresponding to a 64% relative risk reduction (p = 0.015). As expected, bypass and cross-clamp times were longer in the miniAVR group (CPB was 20 minutes longer, p = 0.002), XC was 24.5 min longer, p < 0.0001). Total chest tube drainage was decreased by nearly 300 mL (p < 0.0001).

**Table 1:** 

Variable	Effect Size	P>|z|
Atrial Fibrillation	-26.4%	0.015
MACCE	-8.2%	0.264
CPB (min)	20.0	0.002
XC (min)	24.5	<0.0001
CT Total Output (ml)	-295.8	<0.0001
In-hospital Mortality	-2.0%	0.396
30 day mortality	-1.4%	0.59
Stroke	-2.0%	0.587
Pneumonia	-4.8%	0.067
Takeback to OR	-1.4%	0.448
Intubation Time (hrs)	-2.4	0.2

## Discussion/Conclusion

A dedicated change in practice to miniAVR is safe, associated with improved outcome, is favored by patients and referring providers and may be associated with a significant reduction in post-operative atrial fibrillation.

